# Multidisciplinary Tumor Board Evaluation of Pediatric Patients with Adrenocortical Tumors Across Seven International Centers

**DOI:** 10.3390/cancers17061014

**Published:** 2025-03-17

**Authors:** Maria Riedmeier, Wiebke Schlötelburg, Shipra Agarwal, Ahitagni Biswas, Saniye Ekinci, Martin Fassnacht, Maria C. B. Villares Fragoso, E. Nazli Gonc, Melis Gultekin, Mithat Haliloglu, Vishesh Jain, Manisha Jana, Dominika Janus, Jagdish Prasad Meena, Jessica Munarin, Diclehan Orhan, Jaydira Del Rivero, Rajni Sharma, Gerdi Tuli, Bilgehan Yalcin, Verena Wiegering

**Affiliations:** 1 Department of Pediatrics, Division of Pediatric Hematology, Oncology and Stem Cell Transplantation, University Hospital Würzburg, University of Wuerzburg, Josef-Schneiderstr. 2, 97080 Wuerzburg, Germany; riedmeier_m@ukw.de; 2 Department of Nuclear Medicine, University Hospital Würzburg, University of Wuerzburg, Oberdürrbacherstrasse 6, 97080 Wuerzburg, Germany; schloetelburg_w@ukw.de; 3 Department of Pathology, All India Institute of Medical Sciences, New Delhi 110029, India; drshipra0902@aiims.edu; 4 Department of Radiation Oncology, All India Institute of Medical Sciences, New Delhi 110029, India; dr_ahitagni@yahoo.co.in; 5 Department of Pediatric Surgery, Hacettepe University Faculty of Medicine, 06100 Ankara, Türkiye; 6 Department of Medicine, Division of Endocrinology and Diabetes, University Hospital Würzburg, University of Wuerzburg, Oberdürrbacherstrasse 6, 97080 Wuerzburg, Germany; fassnacht_m@ukw.de; 7 Comprehensive Cancer Centre CCC WERA, University of Wuerzburg Medical Centre, Josef-Schneiderstr. 2, 97080 Wuerzburg, Germany; 8 Disciplina de Endocrinologia e Metabologia Adrenal Unit, Instituto do Câncer do Estado de São Paulo, Hospital das Clinicas, Faculdade de Medicina da Universidade de São Paulo, São Paulo 01246-000, Brazil; maria.villares@hc.fm.usp.br; 9 Department of Pediatric Endocrinology, Hacettepe University Faculty of Medicine, 06100 Ankara, Türkiye; ngonc@hacettepe.edu.tr; 10 Department of Radiation Oncology, Hacettepe University Faculty of Medicine, 06100 Ankara, Türkiye; melisgultekin@hacettepe.edu.tr; 11 Department of Pediatric Radiology, Hacettepe University Faculty of Medicine, 06100 Ankara, Türkiye; 12 Pediatric Surgery, All India Institute of Medical Sciences, New Delhi 110029, India; 13 Radiodiagnosis and Interventional Radiology, All India Institute of Medical Sciences, New Delhi 110029, India; 14 Department of Pediatric and Adolescent Endocrinology, Jagiellonian University Medical College, University Children Hospital, 31-008 Krakow, Poland; dominika.janus@uj.edu.pl; 15 Division of Pediatric Oncology, Department of Pediatrics, Mother & Child Block, All India Institute of Medical Sciences, New Delhi 110029, India; drjagdish2015@aiims.edu; 16 Department of Pediatric Endocrinology, Regina Margherita Children’s Hospital, 10126 Turin, Italy; essica.munarin@unito.it (J.M.); gerdi.tuli@unito.it (G.T.); 17 Department of Pediatrics, University of Turin, 10126 Turin, Italy; 18 Department of Pathology, Hacettepe University Faculty of Medicine, 06100 Ankara, Türkiye; diclehan@hacettepe.edu.tr; 19 Developmental Therapeutics Branch, Center for Cancer Research, National Cancer Institute, National Institutes of Health, Bethesda, MD 20892, USA; jaydira.delrivero@nih.gov; 20 Division of Pediatric Endocrinology, Department of Pediatrics, All India Institute of Medical Sciences, New Delhi 110029, India; 21 Department of Pediatric Oncology, Hacettepe University Faculty of Medicine, 06100 Ankara, Türkiye; yalcinb@hacettepe.edu.tr; 22 KIONET, Pediatric Oncology Network and Bavarian Center for Cancer Research, 97080 Wuerzburg, Germany; 23 Mildred Scheel Early Career Center, University Hospital Wuerzburg, 97080 Wuerzburg, Germany

**Keywords:** pediatric ACC, ring study, reference center, inter-center agreement, guideline, therapy recommendation

## Abstract

Background: The management of rare tumors at reference centers with specialized multidisciplinary tumor boards (MTBs) improves patient survival. No international standardized diagnostic and treatment recommendations for pediatric patients with adrenocortical carcinoma (ACC) exist so far. The aim of this case-based study is to determine how congruent treatment recommendations are in different specialized institutions in different countries. Methods: In this cross-sectional, case-based survey study, five anonymized patient cases, including imaging data of pediatric adrenocortical carcinomas (pACCs), were distributed to seven international specialized centers located in Brazil, Germany, India, Italy, Poland, Turkey, and the USA. The centers were tasked with providing recommendations regarding the treatment sequence and modalities, as well as indicating the level of consensus on these decisions within their MTBs. For each case, the treatment approach recommended by the highest number of centers was recorded to calculate an agreement percentage relative to all treatment recommendations made. The consensus level for each case was determined on a scale of 1 to 10, with higher scores indicating greater agreement among MTB members. Results: A total of five patient cases were discussed across seven MTBs, yielding a total of 34 treatment recommendations. The agreement percentages for the primary therapeutic approach for each case were as follows: primary therapeutic approach: 88.6%; surgical approaches: 51.8%; and systemic chemotherapy recommendations: 53%. Conclusions: This study represents the first case-based analysis of international inter-center agreement on pediatric ACC management. Inter-center agreement regarding treatment allocation and sequencing was low, while the level of consensus within each MTB was high. These findings point to potentially significant and clinically relevant differences in treatment standards across centers, highlighting the need for international collaboration and standardized diagnostic and therapeutic recommendations, especially for rare tumors. Increased case-based exchanges between centers are essential to reduce the substantial discrepancies observed here and to further standardize the management of pediatric patients with ACC.

## Simple Summary

Currently, there are no international guidelines for diagnosing and treating pediatric adrenocortical carcinoma (ACC). This study examines how treatment recommendations vary across specialized centers in different countries. It is the first case-based analysis of an international agreement on pediatric ACC management. The findings reveal significant differences in treatment approaches between centers, even though individual tumor boards within each institution show strong internal consensus. These discrepancies highlight the need for global collaboration and standardized guidelines, particularly for rare cancers. Enhancing case-based discussions between institutions could help unify treatment strategies and improve care for pediatric patients with ACC.

## 1. Introduction

Adrenocortical carcinomas (ACCs) are highly malignant tumors originating from the adrenal cortex. This tumor type is rare in both adults and children [1], with an incidence of 0.2–0.3 cases per 1 million individuals per year in patients under 20 years of age [2,3]. In children, ACC can occur sporadically or as part of hereditary syndromes such as Li–Fraumeni, Beckwith–Wiedemann, and MEN1 [4,5]. For instance, in Southern Brazil, the incidence of pediatric ACC is approximately 15 times higher due to an endemic germline mutation in the TP53 gene (p.R337H) [6,7,8,9]. Research on pediatric ACCs concerning clinical diagnosis, histopathology, and treatment is limited. The few studies available comparing pediatric and adult ACCs suggest significant differences, indicating that knowledge from adult ACCs cannot be directly applied to pediatric cases without careful consideration [10,11,12,13]. In contrast to adult patients, nearly all pediatric patients with ACCs are hormonally active and typically present with clinical features such as virilization, Cushing’s syndrome, or precocious puberty [14,15]. The disease shows peak incidence in infancy and post-puberty [16]. Known risk factors in pediatric patients with ACC include age, tumor size, stage, and operability, as well as specific immunohistopathological criteria such as Ki-67 and certain hormone profiles [17,18].

Surgical resection of the tumor is the primary and most effective treatment; in advanced stages, systemic chemotherapy and mitotane therapy are also employed [19,20,21]. The prognosis is poor, with a 5-year survival rate of less than 40% [16,18]. However, the prognosis is more favorable in infants, with an overall survival rate of approximately 80% for those under 4 years of age [18]. Currently, no effective, established therapy exists for advanced ACCs.

As with many rare pediatric tumor types, international standardized diagnostic or therapeutic guidelines for pediatric ACCs are lacking. Treatment protocols and outcome reports vary widely. In recent years, significant efforts have been made at the European level (ExPERT), Brazilian level (IC-PACT), and international level (ENSAT-PACT) to unite experts, collect international data, and develop improved treatment strategies and recommendations [17,22,23,24,25,26,27].

To evaluate the consistency of care for pediatric patients with ACC across specialized centers worldwide, seven centers were asked to review five constructed, representative pediatric ACC cases within their tumor boards and provide treatment recommendations. The goal was to gather real-world data on current care practices, identify potential differences, and work towards standardizing and improving therapies in the long term.

## 2. Materials and Methods

In this cross-sectional, case-based survey study, we share five anonymized pediatric ACC cases with seven specialized ACC centers located in Brazil, Germany, India, Italy, Poland, Turkey, and the USA. Each case includes details on patient demographics, initial symptoms, imaging results, and pathology findings, which were reviewed in the multidisciplinary tumor board (MTB) meetings at each center. All cases were presented under the assumption that the patients had a good performance status, no significant comorbidities, and a preference for aggressive treatment. The participating centers, all affiliated with ENSAT-PACT, were invited via email in May 2024 and asked to discuss the cases during their MTB sessions. These discussions occurred between May and August 2024. Participants were fully briefed on the study’s purpose, and decision-making was approached as if they were providing a second opinion. Notably, they were not informed about the actual treatments administered or the conclusions reached by other centers.

Centers were subsequently asked to complete a written questionnaire in English for each case (refer to Supplementary File S1). Initially, they were invited to provide their MTB recommendations in an open-ended format. Following this, a multiple-choice question assessed whether additional diagnostic tests were necessary, and if so, participants were asked to specify which ones. Another open-ended question then sought treatment recommendations, assuming no further diagnostics were required. The next section focused on potential therapeutic strategies, encompassing surgery, chemotherapy, radiotherapy, targeted therapies, clinical trial participation, best supportive care, or other options. Each treatment category included detailed follow-up queries: for chemotherapy or targeted therapies, information was requested on the specific agent, dosage, cycle count, and duration; for surgical interventions, details regarding resection extent, wound reconstruction, and preservation of critical structures were needed; and for radiotherapy, specifics on radiation type, dose per fraction, daily fractionation, total dose, and technique were collected. Additionally, the questionnaire addressed the sequencing of proposed therapies, followed by a request for the rationale behind the chosen approach. Specialists were then asked to rate the level of consensus within their MTB on a scale from 1 (lowest) to 10 (highest). The final two questions explored the best alternative treatment strategy and the composition of specialist disciplines present during the MTB discussions.

Responses to the multiple-choice questions were analyzed and summarized descriptively based on the total number of responses. Open-ended answers underwent a structured multi-step analysis: first, all responses were compiled into a single dataset; then, they were categorized, followed by an assessment of the frequency of each category; finally, the findings were interpreted in relation to the respective questions. Any responses that were ambiguous or unrelated to the topic were excluded from the analysis. The results were presented descriptively, reflecting the total number of responses. While abstentions were recorded, they were not included in the descriptive statistical analysis.

The study received approval from the institutional review board (ethics committee of Würzburg; reference number 2024031301).

For each of the five cases, treatment recommendations from the seven centers were classified into three levels of detail. At the most detailed level, recommendations were examined based on the specific treatment modality and the proposed treatment sequence. In the final and most generalized level, pre- and postoperative treatments were merged into a single perioperative category for a more consolidated analysis.

## 3. Results

### 3.1. Patient Characteristics

**Figure 1:**
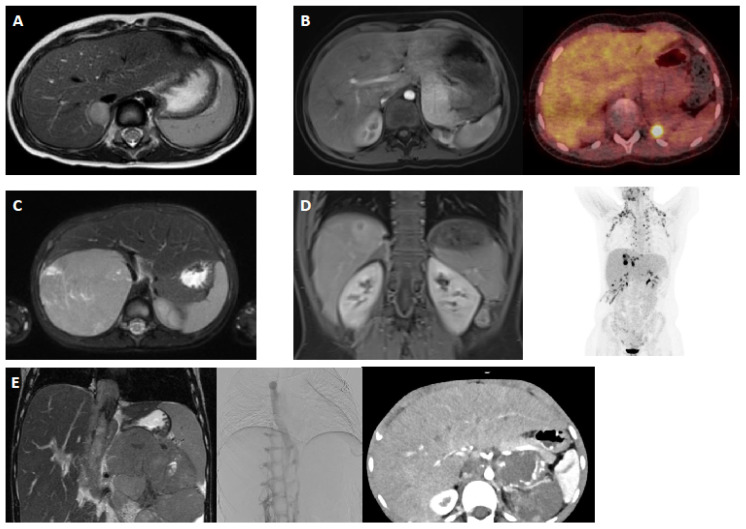
Image examples of the five patient cases: (**A**) An axial T2w image of the right-sided adrenocortical carcinoma of patient 1. (**B**) An axial T1w image with fat saturation post contrast and axial F18-FDG-PET/CT fusion of the left-sided adrenocortical carcinoma of patient 2. (**C**) An axial T2w image with fat saturation of the right-sided adrenocortical carcinoma of patient 3. (**D**) A coronal T1w image post contrast and whole-body MIP of the right-sided adrenocortical carcinoma of patient 4. (**E**) A coronal T2w image, cavography (with obstruction of VCI) and axial CT scan post contrast of the left-sided adrenocortical carcinoma of patient 5.

**Figure 2:**
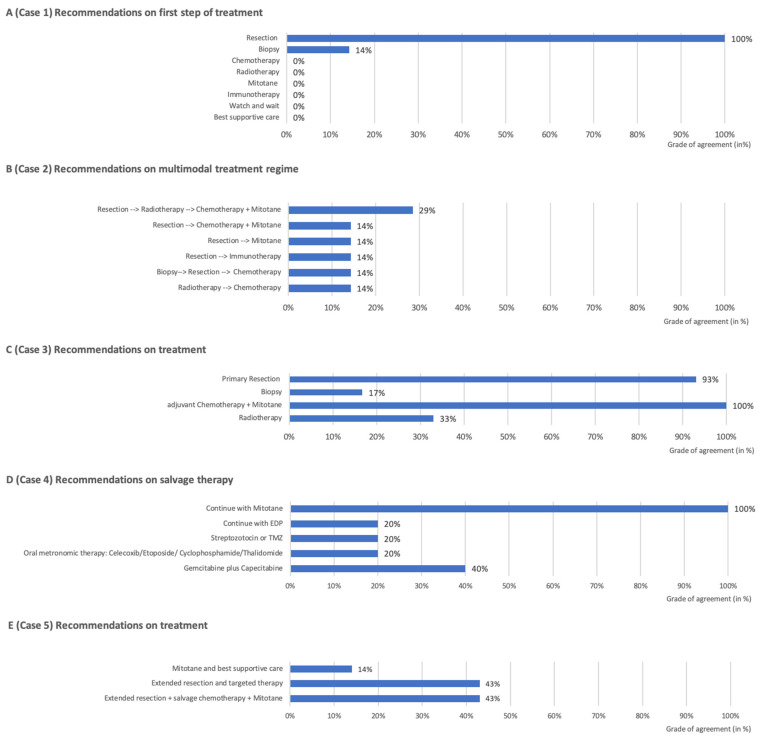
(**A**–**E**) Treatment recommendations of the 5 clinical cases (1–5; **A**–**E**).

Patient characteristics and radiological images are summarized in Table 1 and Figure 1. A total of five cases were evaluated by seven MTBs, resulting in 34 treatment recommendations (Table 2 and Figure 2). All MTB meetings involved at least four specialties: radiology (including neuroradiology), (pediatric) oncology, (pediatric) endocrinology, and surgery. In four centers, pathology was also included.

### 3.2. Case 1: Localized Right-Sided Adrenocortical Tumor with Androgen Secretion, Initial Diagnosis (For Case 1 Details See Table 1 and Figure 1A)

Out of the seven responding centers, all (100%) recommended surgical intervention as the primary therapeutic approach (see Figure 2A). None of the centers suggested a watch-and-wait strategy or a neoadjuvant approach. However, one center (14%) preferred performing a biopsy prior to resection. Regarding the surgical technique, two out of seven centers (29%) recommended laparotomy, another two (29%) favored a laparoscopic approach, and the remaining three centers (43%) did not specify a preferred surgical method. Lymph node dissection was recommended by all centers.

For further diagnostic workup, all centers recommended genetic counseling (100%) and additional staging with PET-CT (100%). One center (14%) suggested including a brain MRI in the initial staging. Pre-surgical endocrine evaluation was recommended by six out of seven centers (86%). Specifically, five of these six centers (83%) recommended a 24 h urine sample for steroid profiling, and the same proportion (83%) advised measuring 17-hydroxyprogesterone (17-OHP). Four centers (66%) suggested assessing serum levels of androstenedione, testosterone, follicle-stimulating hormone (FSH), luteinizing hormone (LH), estradiol, adrenocorticotropic hormone (ACTH), and cortisol, as well as dehydroepiandrosterone sulfate (DHEAS). Additionally, two centers (33%) recommended a dexamethasone suppression test, and two centers (33%) proposed a carpogram.

Systemic treatment and postoperative radiotherapy were recommended only based on histologic findings and the extent of resection. The average consensus level across centers was 9.8.

### 3.3. Case 2: Second Local Relapse of Stage IV Left-Sided Adrenocortical Carcinoma with Androgen Secretion (For Case 2 Details, See Table 1 and Figure 1B)

Of the seven responding centers, all provided treatment recommendations. Six of the seven MTBs (86%) recommended surgery as the primary therapeutic approach, while one center (14%) favored a primary radiotherapeutic approach. No center suggested a neoadjuvant approach. Among those recommending surgery, four out of six centers (57%) advocated open laparotomy with regional lymph node dissection, one center preferred a laparoscopic approach, and one center recommended a preliminary biopsy (see Figure 2B).

Treatment recommendations included the following multimodal sequences: two centers (29%) recommended resection followed by radiotherapy, and then chemotherapy with mitotane; one center (14%) recommended resection followed by chemotherapy with mitotane; one center (14%) recommended resection followed by mitotane alone; one center (14%) suggested resection followed by targeted biological therapy with cabozantinib; one center (14%) recommended biopsy followed by resection and chemotherapy; and one center (14%) proposed radiotherapy followed by chemotherapy. Chemotherapeutic regimens discussed included EDP (etoposide, doxorubicin, and cisplatin), temozolomide, and gemcitabine plus capecitabine. In total, four centers (57%) considered radiotherapy as part of the treatment: one in a neoadjuvant setting, two in an adjuvant setting, and one in the case of incomplete resection (see Figure 2B).

For further diagnostic workup, additional staging with PET-CT was recommended by six out of seven centers (86%). All centers (100%) advised conducting an endocrine evaluation prior to the surgical approach.

The average consensus level across the centers was 9.

### 3.4. Case 3: Right-Sided Adrenocortical Tumor of Stage III with Androgen Secretion, Initial Diagnosis (For Case 3 Details, See Table 1 and Figure 1C)

In total, six out of the seven centers provided treatment recommendations (see Figure 2C). All centers (6/6) favored surgery as the initial treatment step. Among them, four centers (67%) preferred open laparotomy with lymph node dissection, while one center (16%) opted for a laparoscopic approach. The remaining center (16%) did not specify a preferred surgical method. Additionally, one center (16%) recommended performing a biopsy prior to resection.

All centers voted for adjuvant chemotherapy with EDP or EDP-like regimens plus mitotane due to the stage III tumor classification. Additionally, adjuvant local radiotherapy was discussed in two MTBs (2/6, 33%).

The most common diagnostic recommendations included a diagnostic approach using FDG-PET (100%), an endocrine workup (100%), and genetic counseling (83%). The endocrine evaluation was recommended as a baseline and included serum measurements of LH, FSH, ACTH, testosterone, androstenedione, estradiol, basal cortisol, 17-hydroxyprogesterone (17 OHP), and DHEAS (6/6, 100%), along with a 24 h urine collection for steroid profiling (4/6, 67%).

The average consensus level among the MTBs across all centers was 9.8.

### 3.5. Case 4: Stage IV Left-Sided Adrenocortical Carcinoma, Cortisol-Secreting, with Multiple Metastases and Multiple Disease Progressions (For Case 4 Details, See Table 1 and Figure 1D)

The seven centers provided seven different treatment recommendations (see Figure 2D). All centers acknowledged that the patient has a very poor prognosis and that palliative care should be discussed with the family (7/7; 100%). None of the centers recommended surgery, except for symptomatic relief.

Five out of seven centers (71%) proposed salvage therapies, which included two centers (40%) recommending gemcitabine plus capecitabine; one center (20%) suggesting oral metronomic therapy with celecoxib, etoposide, cyclophosphamide, and thalidomide; one center (20%) proposing streptozotocin or temozolomide (TMZ); one center (20%) advising continuation of EDP; and one center recommending continuing with mitotane. Additionally, two out of seven centers (29%) discussed the option of palliative radiotherapy. All multidisciplinary tumor boards recommended continuing mitotane treatment, and medications such as ketoconazole or metyrapone were suggested to improve symptom control.

An average degree of consensus of 9.0 was reached.

### 3.6. Case 5: Adrenocortical Carcinoma (Left Side), Stage IV, Androgen-Producing After Neoadjuvant Therapy (For Case 5 Details, See Table 1 and Figure 1E)

All seven centers provided treatment recommendations (see Figure 2E). Following neoadjuvant chemotherapy, six out of seven MTBs (86%) recommended an extended tumor resection at an expert center, including lymph node dissection. One center (14%) advised continuing mitotane therapy and only considering surgery in the event of disease progression.

In cases of progression or incomplete resection, three out of seven centers (43%) recommended salvage therapy, which included two centers proposing gemcitabine plus capecitabine and one center suggesting oral metronomic therapy with celecoxib, etoposide, cyclophosphamide, and thalidomide. Additionally, three out of seven centers (43%) recommended targeted therapies, such as pembrolizumab or cabozantinib, while one center (14%) advised providing best supportive care. Mitotane maintenance therapy after surgery was recommended by five out of seven centers (71%) for a duration of at least 3 to 5 years.

The average consensus level among the multidisciplinary tumor boards across all centers was 9.2.

### 3.7. Summative Analysis of Agreement for All Cases

The agreement among the MTBs regarding the first therapeutic step demonstrated a high level of consensus at 88.6%. In the detailed analysis of surgical recommendations, the average agreement percentage across all five cases was 51.8%. For systemic chemotherapy recommendations, the mean agreement percentage was slightly lower at 53%. Furthermore, the average consensus level within each center across all five cases was 9.4.

## 4. Discussion

In this study, we present the first analysis of inter-center agreement across seven international expert centers for pediatric ACC based on five common pediatric ACC cases. Overall, the inter-center agreement on treatment allocation was low, while the consensus within each center was very high.

As with other rare tumors, there is a notable lack of large international prospective studies to inform evidence-based treatment recommendations for pediatric ACC. Consequently, many therapies are adapted from other tumor types or from adult treatment protocols [28]. Treatment choices and decisions are often influenced by personal experience and country-specific protocols and standards. In recent years, various international networks (ENSAT-PACT, ExPERT, and IC-PACT) have made significant efforts to bring together experts, analyze existing retrospective data, and conduct prospective studies to better understand therapy efficacy and identify potential risk factors [17,21,22,23,24,25,26,27]. These efforts aim to standardize and optimize treatment approaches for pediatric ACC over the long term.

Current data indicate that while knowledge from adult cases provides insights, it cannot be applied uncritically to pediatric patients as tumor behavior in pediatric cases can differ significantly [10,16]. Additionally, due to the larger patient populations in adult studies, promising findings and successful approaches should also be tested specifically in pediatric settings. Experience is particularly crucial in the surgical domain, as numerous studies have demonstrated that centralizing complex tumor interventions leads to significantly fewer complications and improved overall survival [29,30]. However, implementing this in routine practice is challenging, especially for rare diseases with limited specialists.

In this tumor board evaluation, our goals were to collect real-world data from experienced expert centers to examine the variability in diagnostic and therapeutic approaches and to identify ways to enhance and harmonize treatment standards.

Several factors may underlie the notable variation observed across centers in this study. First, there is a scarcity of clinical data, especially regarding optimal treatment sequencing, with limited head-to-head studies available to compare different approaches. It may also be argued that the exact order of treatment steps may not result in significant clinical differences; for instance, recommendations for pre- or postoperative chemotherapy could be generalized under “perioperative chemotherapy”, which could help minimize apparent variability between centers.

Another contributing factor may be center-specific practices and routines that have evolved over time, favoring certain treatment combinations or sequences without definitive evidence to support or refute these preferences. While finding scientific support for this hypothesis is challenging, the authors anticipated this phenomenon, recognizing center-specific routines as a common reality in clinical practice. This is also reflected in the high level of internal consensus within each center, with an average consensus rating of 9.0, indicating a strong alignment within individual centers on their own treatment protocols. This observation underscores the need for international registries and standardized therapy protocols to ensure uniform standards and quality of care for all patients.

The study demonstrates that current treatment approaches rely primarily on the experience and expertise of individual centers and are only minimally evidence based. This reliance is partly due to the lack of representative studies, especially in pediatric populations, and also reflects an absence of unified expert recommendations. In other areas lacking a robust evidence base, consensus recommendations have been successfully developed through DELPHI processes. This underscores the importance of uniting stakeholders and experts within collaborative networks for rare diseases to optimize therapy comprehensively—addressing fundamental scientific, translational, and clinical aspects—and to make meaningful advancements in treatment outcomes.

It can be assumed that there are significant country-specific and socio-economic differences that are difficult to distinguish in detail here. Nevertheless, with the exception of mitotane, the recommended treatments primarily involve surgical and conventional chemotherapeutic approaches, which should be available as the standard of care in all of these countries. Against this backdrop, internally developed therapy guidelines, particularly for rare diseases, appear highly relevant to ensuring that all patients receive adequate basic treatment.

Regarding mitotane treatment, all members of the MTBs agreed and it should be explicitly emphasized that the absolute dosage is less important than the therapeutic level achieved in terms of effectiveness [23,31,32]. Therefore, the dosage should be individually adjusted and monitored for each patient, and the duration of therapy should be based on the goal of maintaining the therapeutic level.

A critical tool for diagnosis mentioned in the questionnaire is tumor biopsy, but international guidelines for pediatric ACC are lacking. Given the significant risk of tumor cell spread and even tumor rupture, current adult treatment guidelines recommend biopsy only for patients with primarily inoperable tumors that show ambiguous or no hormone secretion in order to obtain a reliable diagnosis [28]. Regarding the risk of rupture, studies on other carcinomas have demonstrated that newer, radiologically guided procedures are superior to open surgical techniques [33,34,35]. This is likely to apply to ACC as well. However, the tissue yield from these procedures is significantly lower, which makes molecular genetic analysis more challenging. Therefore, the choice of technique must always be made on an individual basis, carefully weighing the benefits and risks for each patient.

The strengths of our study include its novelty and validity as it is the first to systematically compare multidisciplinary and inter-center treatment recommendations across various common clinical scenarios of pediatric ACC. This study provides real-world data in an international context, offering a unique insight into clinical practice. However, several limitations should be considered. First, the sample size of five cases is relatively small; a larger and more diverse set of cases would more accurately reflect clinical reality. This limited sample size also restricts the ability to perform a robust statistical analysis of the agreement levels among centers. Second, the survey used in this study was not a validated instrument; instead, it was designed to address practical, specific questions on managing the presented cases. Third, participating specialists in the MTBs were informed in advance of the study’s aims and knew that their responses would be analyzed collectively, without direct clinical consequences for the patients, which may have influenced their decision-making. Finally, the information provided to the MTBs was limited to what was included in each case, unlike real clinical settings where ambiguous findings often prompt additional diagnostics for more informed decision-making. Notably, requests for further diagnostics were only made in a few cases, as each case description included only a brief summary of the key information.

## 5. Conclusions

In summary, this study presents the first case-based analysis of international inter-center agreement on the management of pediatric ACC. While inter-center agreement on treatment allocation and sequencing was low, the degree of consensus within each MTB was high. These findings suggest potentially significant and clinically relevant differences in treatment standards across centers, underscoring the need for international collaboration and standardized diagnostic and therapeutic guidelines, particularly for rare tumors. Case-based exchanges between centers are essential to reduce the considerable discrepancies highlighted in this study and to further standardize the management of pediatric patients with ACC.

## Figures and Tables

**Table 1:** Patient characteristics of the five clinical cases of pediatric ACCs, including the exact diagnosis, clinical course, pathological and genetic findings, radiological results, further diagnostic evaluations, and the questions posed to the MTBs. ^1^ This table includes only the pathological, genetic, and radiological details that were available for the tumor board evaluation.

Case	Patient Characteristics			Diagnosis	Clinical Course	Pathology ^1^	Genetics ^1^	**Radiology ^1^**	**Further** **diagnosis**	**Question**
Number	Age (in Years)	Sex	Family History							
1	3.8	Female	Without events	Adrenocortical tumor (right side), androgen-secreting	-Clitoral hypertrophy detected 10 months ago during check-up; -Inconspicuous female chromosome set -Endocrine workup: marked hyperandrogenemia; DHEAS-B value: 4.870 µg/L; normal FSH, LH, estradiol, testosterone, ACTH, and cortisol levels; AGS excluded -Ultrasound: suspicious for focal nodular hyperplasia (FNH) of liver -Further staging: no pathological findings	Not complete	Not complete	MRI: adrenal gland tumor right CT Thorax: no filiae (see Figure 1A)	Mild development delay	Primary diagnosis -treatment recommendations
2	6.9	Female	Without events	Adrenocortical carcinoma (left side) initially stage 2, relapse stage IV, 5.4 × 5.2 × 5.2 cm, androgen-secreting	-04/21: First diagnosis -01/22: Relapse (local, lungs, kidney) -Previous oncological therapy: 04/21: Adrenalectomy (R0 resection) 01/22: Surgery on 4.9 cm lung metastasis 02/22: Partial kidney resection with 2 cm ACC metastasis 04/22: Partial renal re-resection with confirmed metastasis 03–09/22: Six cycles of chemotherapy (EDP) per GPOH-MET registry Since 03/22: Mitotane therapy, levels in target range since 09/22 -Follow up 09/2023: tumor left adrenal loge	Wieneke Score: 4 Ki-67 expression: 20%, focal areas 40%	No germline mutation detected INFORM study inclusion: no molecular target identified Borderline findings: AURKC, TYK2, and GDF overexpression	MRI: left adrenal loge CT Thorax: no filiae (see Figure 1B)	None	Local relapse of left adrenal gland 9/23 -treatment recommendations
3	3.1	Female	Without events	Adrenocortical tumor (right side) stage III androgen-secreting	-Clinical signs of pubertal hair development and clitoral hypertrophy over few weeks	Not complete	Not complete	MRI: adrenal tumor CT Thorax: no filiae (see Figure 1C)	None	Primary diagnosis -treatment recommendations
4	12.8	Female	Without events	Adrenocortical carcinoma (left side), stage IV, cortisol-secreting, metastases: lymph nodes, liver, pulmonary	-02/23: First diagnosis -02–08/23: Eight cycles of chemotherapy (EDP) + mitotane (per GPOH-MET registry) -> partial response -10/23: Tumor resection with laparotomic adrenalectomy (right side), tumor mass reduction -11/23: Tumor progression; adjuvant chemotherapy (2 cycles of EDP) plus mitotane	Wieneke Score: 6 Ki-67 expression: 20%, focal areas 40%	No germline mutation detected INFORM study inclusion: no molecular target identified Borderline findings: FGFR1, VEGFA	MRI/FDG-PET: progression of metastases (liver and pulmonal) (see Figure 1D)	Epilepsy	Progression of metastases (liver and pulmonal) -further treatment recommendations
5	5.4	Female	Without events	adrenocortical carcinoma (left side), stage IV Tumor size: 16 × 11 × 16 cm, androgen-secreting	-02/23: First diagnosis with clinical signs of precocious puberty Infiltration: Vena cava, thrombus in right atrium, pulmonary filiae -02–08/23: Eight blocks of chemotherapy (EDP) according to GPOH-MET registry + mitotane	Not available	Li–Fraumeni syndrome	MRI/ CT Thorax: partial response (see Figure 1E)	Li–Fraumeni syndrome Arterial hypertension AV block (first degree), mild QTc elongation	Staging at end of intensive chemotherapy further treatment recommendations (surgery? immunotherapy? mitotane only?)

**Table 2:** Diagnostics and therapy for the five patient cases. A summary of diagnostic and treatment decisions for cases 1–5 containing additional diagnostics, surgery, systemic treatment, mitotane, radiotherapy, and other therapies, along with the percentage of recommendation among the respective centers.

Case	Additional Diagnostics	Surgery	Systemic Treatment	Mitotane	Radiotherapy	Others
1	PET-CT (100%) cranial MRI (17%) endocrine workup (86%)	Primary surgical approach (100%)	Systemic treatment in dependency of histology and surgical outcome (100%)			Genetic counseling (100%)
2	PET-CT (86%) endocrine workup (100%)	Primary surgical approach (86%)	Systemic adjuvant therapy (100%) chemotherapy (71%) Mitotane alone (14%) Mitotane plus chemotherapy (42%)		Consideration of radiotherapy (67%) (neoadjuvant 14%, adjuvant 43%)	Adjuvant targeted therapy with cabozantinib (14%)
3	PET-CT (100%) endocrine workup (100%)	Primary surgical approach (100%)	Adjuvant EDP-like chemotherapy (100%)	Mitotane treatment (100%)	Adjuvant radiotherapy in addition to systemic treatment (33%)	Genetic counseling (100%)
4	No additional diagnostics	Not recommended	Salvage therapy (71%)	Continuation of mitotane (100%)	Additional radiotherapy (29%)	Palliative setting (100%) Ketoconazole/metyrapone symptomatically
5	No additional diagnostics	Extended tumor resection (86%)	Neoadjuvant chemotherapy plus mitotane (100%), mitotane maintenance therapy (71%)		Not recommended	Adjuvant targeted/ biological therapy (43%)

## Data Availability

The MRI scans and the responses from the centers to the questionnaire are available from the authors upon request.

## Supplementary Materials

The following supporting information can be downloaded at: https://www.mdpi.com/article/10.3390/cancers17061014/s1, File S1: Questionnaire provided to the participants of the tumor board evaluation.
