# Supplementary material for: Multidisciplinary Tumor Board Evaluation of Pediatric Patients with Adrenocortical Tumors Across Seven International Centers
Source: Cancers (Basel). 2025 Mar 17;17(6):1014. doi: 10.3390/cancers17061014 (PMC11940591; doi:10.3390/cancers17061014)
Supplement: Supplementary file 1 [file cancers-17-01014-s001.zip › cancers-3500227-supplementary.pdf]

Supplemental File S1_ Questionnaire provided to the participants of the tumor board evaluation

Study: Tumor conference trial pediatric ACC

As a tumor conference, please provide a recommendation for the further procedure or a next therapy proposal as free text and as a multiple choice option with multiple selections - assuming that the patient has a maximum desire for therapy and no limiting comorbidities.

Please provide a brief clinical rationale for your decision, e.g. referring to scientific publications or previous clinical experience.

Deadline: End of May

Name of the centre completing the form: ______________________________

Case 1

**Patient characteristics**	• Age 3 10/12<br>• Sex: female<br>• Family history: without events
**Diagnosis**	• Adrenal gland Tumor right
**Clinical Course**<br><br>**Question**	• Clitoral hypertrophy detected 10 months ago as part of a check-up<br>• inconspicuous female chromosome set and marked hyperandrogenaemia with a DHEAS-B value of DHEAS-B value of 4,870 µg/l with inconspicuous values for FSH, LH, oestradiol, testosterone, ACTH and cortisol<br>• Exclusion of an AGS<br>• Ulltrasound: suspicious of FNH of the liver<br>• MRI: Adrenal gland Tumor right<br>• Further staging without pathologic results<br><br>**Primary diagnosis- treatment recommendation**
**Pathology**	Not done
**Radiology**	MRI: pictures are uploaded<br>CT Thorax: no filiae
**Other diagnosis**	• Mild developmental delay<br>• No other diagnosis
**Please enter your tumour conference recommendation as free text**	
**Further diagnostics necessary?**	☐ No<br><br>☐ **Yes (multiple selections are possible):**<br><br>☐ Repeat follow-up imaging — ☐ PET-CT<br>☐ Biopsy — ☐ others<br>☐ Genetics<br>☐ recommended<br>☐ obligatory<br>☐ not necessary<br><br>Comments (free text):
**Recommended endocrine workup (free text)**	
**What are your treatment recommendations at the present time and without further diagnostics (free text)?**	
	☐ Resection (=OP). If yes, please specify (multiple selection):

<table>
<tr><td rowspan="9">What are your treatment recommendations at the present time and without further diagnostics (multi-ple choice possible)?</td><td>☐ preferred laparoscopic
☐ preferred Laparotomy
☐ lymphnode dissection
☐ Biopsy
☐ preferred sonographic
☐ preferred Laparoscopic
☐ Watch and wait
☐ Others
Comments: ______________________</td></tr>
<tr><td>☐ Chemotherapy (=CTX)
• Active substance:
• Dose:
• Number of cycles:
• Duration per cycles:</td></tr>
<tr><td>☐ Radiotherapy (=RT)
• Type of radiation (photons, protons, etc.):
• Dose per Fraction:
• Fraction per day:
• Total dose:
• Technic:</td></tr>
<tr><td>☐ Mitotane
• Target serum level:
• Initial dose:
• Duration of treatment:</td></tr>
<tr><td>☐ Targeted therapy (=TT)
• Active substance:
• Dose:
• Number of doses:</td></tr>
<tr><td>☐ Best supportive care</td></tr>
<tr><td>☐ Inclusion in the following study/Registry:</td></tr>
<tr><td>☐ Other suggestions:</td></tr>
<tr><td></td></tr>
<tr><td>Multimodal therapy:

If you have recommended a multimodal therapy, please indicate the planned se-quence</td><td>- - -</td></tr>
</table>

<table>
<tr><td>What is your rationale for the procedure (free text) ?</td><td colspan="2"></td></tr>
<tr><td>What was the consensus of all parties involved in this decision? (from 1(low)-10 (high))</td><td colspan="2">1 ☐ 2 ☐ 3 ☐ 4 ☐ 5 ☐ 6 ☐ 7 ☐ 8 ☐ 9 ☐ 10 ☐</td></tr>
<tr><td>Best alternative procedure (free text) ?</td><td colspan="2"></td></tr>
<tr><td rowspan="9">Specialities present at the tumour conference meeting (multiple choice possible)</td><td>☐ General and visceral surgery</td><td>☐ Pediatric surgery</td></tr>
<tr><td>☐ Radiotherapy</td><td>☐ ped. Oncology</td></tr>
<tr><td>☐ Pathology</td><td>☐ adult Oncology</td></tr>
<tr><td>☐ Radiology (incl. Neurorad.)</td><td>☐ Nuclearmedicine</td></tr>
<tr><td>☐ ped. Endocrinology</td><td>☐ adult Endocrinology</td></tr>
<tr><td>☐</td><td>☐</td></tr>
<tr><td>☐</td><td>☐</td></tr>
<tr><td>☐ Urology</td><td>☐</td></tr>
<tr><td>☐ Psychooncology</td><td>☐ social workers</td></tr>
</table>

**Case 2**	
**Patient characteristics**	• Age 6 11/12<br>• Sex: female<br>• Family history: without events
**Diagnosis**	• Adrenal gland Tumor left
**Clinical Course**	• Adrenal carcinoma left diagnosed in 4/21<br>5.4x5.2x5.2cm Tumor<br>Androgen-producing Tumor<br>• Recurrence pulmonary, kidney, local 1/22<br><br>• Previous oncological therapy (OP, RT, CT)<br>Adrenalectomy on 1.4.21 (R0 resection)<br>25.1.22: Operation on a 4.9 cm lung metastasis<br>22.2.22partial kidney resection with 2cm ACC metastasis<br>4/22 partial renal re-resection with evidence of metastasis<br><br>o 6 blocks of chemotherapy according to GPOH-MET registry from 3/22 -12.9.22 (Cisplatin, Eto, Doxo)<br>o Mitotane therapy since 3/22, levels in target range for the first time in September 22
**Question**	**Local recurrence left adrenal gland 9/23- therapy option?**
**Pathology**	• Wieneke Score: 4<br>• Li Fraumeni syndrome was excluded<br>• Mol.-path. of the tumor: no molecular target, borderline: AURKC overexpression, TYK2 overexpression, GDF" overexpression<br>Ki67 expression: 20%, focal 40%
**Radiology**	MRI: pictures are uploaded<br>CT Thorax: no filiae
**Other diagnosis**	• Mild developmental delay<br>• No other diagnosis
**Please enter your tumour conference recommendation as free text**	
**Further diagnostics neces-sary?**	☐ No<br><br>**☐ Yes (multiple choice is possible):**<br><br>☐ Repeat follow-up imaging ☐ PET-CT<br>☐ Biopsy ☐ others<br>☐ Genetics

<table>
<tr><td rowspan="2"></td><td>☐ recommended
☐ obligatory
☐ not necessary</td></tr>
<tr><td>Comments (free text):</td></tr>
<tr><td>Recommended endocrine workup (free text)</td><td></td></tr>
<tr><td>What are your treatment recommendations at the present time and without further diagnostics (free text)?</td><td></td></tr>
<tr><td rowspan="6">What are your treatment recommendations at the present time and without further diagnostics (multiple choice possible)?</td><td>☐ Resection (=OP). If yes, please specify (multiple selection):
☐ preferred laparoscopic
☐ preferred Laparotomy
☐ lymphnode dissection
☐ Biopsy
☐ preferred sonographic
☐ preferred Laparoscopic
☐ Watch and wait
☐ Others
Comments:</td></tr>
<tr><td>☐ Chemotherapy (=CTX)
• Active substance:
• Dose:
• Number of cycles:
• Duration per cycles:</td></tr>
<tr><td>☐ Radiotherapy (=RT)
• Type of radiation (photons, protons, etc.):
• Dose per Fraction:
• Fraction per day:
• Total dose:
• Technic:</td></tr>
<tr><td>☐ Mitotane
• Target serum level:
• Starting Dose:
• Duration of treatment:</td></tr>
<tr><td>☐ Targeted therapy (=TT)
• Active substance:
• Dose:
• Number of doses:</td></tr>
<tr><td>☐ Best supportive care</td></tr>
</table>

<table>
<tr><td rowspan="2"></td><td colspan="2">☐ Inclusion in the following study/Registry:</td></tr>
<tr><td colspan="2">☐ Other suggestions:</td></tr>
<tr><td>Multimodal therapy:

If you have recommended a multimodal therapy, please indicate the planned sequence</td><td colspan="2">- - -</td></tr>
<tr><td>What is your rationale for the procedure (free text) ?</td><td colspan="2"></td></tr>
<tr><td>What was the consensus of all parties involved in this decision? (from 1(low)-10 (high))</td><td colspan="2">1 ☐ 2 ☐ 3 ☐ 4 ☐ 5 ☐ 6 ☐ 7 ☐ 8 ☐ 9 ☐ 10 ☐</td></tr>
<tr><td>Best alternative procedure (free text) ?</td><td colspan="2"></td></tr>
<tr><td rowspan="9">Specialities present at the tumour conference meeting (multiple choice possible)</td><td>☐ General and visceral surgery</td><td>☐ Pediatric surgery</td></tr>
<tr><td>☐ Radiotherapy</td><td>☐ ped. Oncology</td></tr>
<tr><td>☐ Pathology</td><td>☐ adult Oncology</td></tr>
<tr><td>☐ Radiology (incl. Neurorad.)</td><td>☐ Nuclearmedicine</td></tr>
<tr><td>☐ ped. Endocrinology</td><td>☐ adult Endocrinology</td></tr>
<tr><td>☐</td><td>☐</td></tr>
<tr><td>☐</td><td>☐</td></tr>
<tr><td>☐ Urology</td><td>☐</td></tr>
<tr><td>☐ Psychooncology</td><td>☐ social workers</td></tr>
</table>

<table>
<tr><td colspan="3">Case 3</td></tr>
<tr><td>Patient characteristics</td><td colspan="2">• Age 3 years
• Sex: female
• Family history: without events</td></tr>
<tr><td>Diagnosis</td><td colspan="2">• Adrenal gland Tumor right</td></tr>
<tr><td>Clinical Course

Question</td><td colspan="2">• Adrenal carcinoma right
9x7x8cm Tumor
Androgen-producing Tumor

Diagnosed by clinical signs of pubestal hair and clitoral hypertrophy for a few weeks
Initial diagnosis- treatment recommendation</td></tr>
<tr><td>Pathology</td><td colspan="2">Not done yet</td></tr>
<tr><td>Radiology</td><td colspan="2">MRI: pictures are uploaded
CT Thorax: no filiae</td></tr>
<tr><td>Other diagnosis</td><td colspan="2">• No other diagnosis</td></tr>
<tr><td>Please enter your tumour conference recommendation as free text</td><td colspan="2"></td></tr>
<tr><td rowspan="6">Further diagnostics necessary?</td><td colspan="2">☐ No</td></tr>
<tr><td colspan="2">☐ Yes (multiple choice is possible):</td></tr>
<tr><td>☐ Repeat follow-up imaging</td><td>☐ PET-CT</td></tr>
<tr><td>☐ Biopsy</td><td>☐ others</td></tr>
<tr><td>☐ Genetics
☐ recommended
☐ obligatory
☐ not necessary</td><td></td></tr>
<tr><td colspan="2">Comments (free text):</td></tr>
<tr><td>Recommended endocrine workup (free text)</td><td colspan="2"></td></tr>
<tr><td>What are your treatment recommendations at the present time and without further diagnostics (free text)?</td><td colspan="2"></td></tr>
<tr><td>What are your treatment recommendations at the present time and without further</td><td colspan="2">☐ Resection (=OP). If yes, please specify (multiple selection):
☐ preferred laparoscopic
☐ preferred Laparotomy
☐ lymphnode dissection</td></tr>
</table>

**diagnostics (multiple choice possible)?**	☐ Biopsy<br>☐ preferred sonographic<br>☐ preferred Laparoscopic<br>☐ Watch and wait<br>☐ Others<br>Comments: ________________
	☐ Chemotherapy (=CTX)<br>• Active substance:<br>• Dose:<br>• Number of cycles:<br>• Duration per cycles:
	☐ Radiotherapy (=RT)<br>• Type of radiation (photons, protons, etc.):<br>• Dose per Fraction:<br>• Fraction per day:<br>• Total dose:<br>• Technic:
	☐ Mitotane<br>• Target serum level:<br>• Starting Dose:<br>• Duration of treatment:
	☐ Targeted therapy (=TT)<br>• Active substance:<br>• Dose:<br>• Number of doses:
	☐ Best supportive care
	☐ Inclusion in the following study/Registry:
	☐ Other suggestions:
**Multimodal therapy:**<br><br>**If you have recommended a multimodal therapy, please indicate the planned sequence**	- - -
**What is your rationale for the procedure (free text) ?**	

<table>
<tr><td rowspan="1">What was the consensus of all parties involved in this decision? (from 1(low)-10 (high))</td><td colspan="2">1 ☐ 2 ☐ 3 ☐ 4 ☐ 5 ☐ 6 ☐ 7 ☐ 8 ☐ 9 ☐ 10 ☐</td></tr>
<tr><td>Best alternative procedure (free text) ?</td><td colspan="2"></td></tr>
<tr><td rowspan="9">Specialities present at the tumour conference meeting (multiple choice possible)</td><td>☐ General and visceral surgery</td><td>☐ Pediatric surgery</td></tr>
<tr><td>☐ Radiotherapy</td><td>☐ ped. Oncology</td></tr>
<tr><td>☐ Pathology</td><td>☐ adult Oncology</td></tr>
<tr><td>☐ Radiology (incl. Neurorad.)</td><td>☐ Nuclearmedicine</td></tr>
<tr><td>☐ ped. Endocrinology</td><td>☐ adult Endocrinology</td></tr>
<tr><td>☐</td><td>☐</td></tr>
<tr><td>☐</td><td>☐</td></tr>
<tr><td>☐ Urology</td><td>☐</td></tr>
<tr><td>☐ Psychooncology</td><td>☐ social workers</td></tr>
</table>

<table>
<tr><td colspan="3">Case 4</td></tr>
<tr><td>Patient characteristics</td><td colspan="2">• Age 12 9/12
• Sex: female
• Family history: without events</td></tr>
<tr><td>Diagnosis</td><td colspan="2">• Adrenal gland Tumor left</td></tr>
<tr><td>Clinical Course

Question</td><td colspan="2">• ACC right Stage IV diagnosed 2/23
Metastasis lymphnode, liver and pulmonal
• Tumor-associated Cushing-Syndrom by cortison production

• Therapy:
o Chemotherapy according to GPOH-MET Registry plus Mitotane – 8cycles 2-8/23 , partial response
o Open Adrenalectomy right, tumor mass reduction 10/23
o Progression 11/23
o Adjuvant Chemotherapy: 2 cycles EDP plus Mitotane
o
Acute: progression of metastasis liver and pulmonal- further therapy?</td></tr>
<tr><td>Pathology</td><td colspan="2">• Wieneke Score: 6
• Li fraumeni sydrome was excluded
• Mol.-path. of tumor: no molecular target, borderline: FGFR1, VEGFA
Ki67 expression: 20%, focal 40%</td></tr>
<tr><td>Radiology</td><td colspan="2">MRI: pictures are uploaded
FGD-PET</td></tr>
<tr><td>Other diagnosis</td><td colspan="2">• epilepsia
• CPS are excluded</td></tr>
<tr><td>Please enter your tumour conference recommendation as free text</td><td colspan="2"></td></tr>
<tr><td rowspan="5">Further diagnostics necessary?</td><td colspan="2">☐ No</td></tr>
<tr><td colspan="2">☐ Yes (multiple choice is possible):</td></tr>
<tr><td>☐ Repeat follow-up imaging</td><td>☐ PET-CT</td></tr>
<tr><td>☐ Biopsy</td><td>☐ others</td></tr>
<tr><td>☐ Genetics
☐ recommended
☐ obligatory
☐ not necessary</td><td></td></tr>
</table>

	Comments (free text):
**Recommended endocrine workup (free text)**	
**What are your treatment recommendations at the present time and without further diagnostics (free text)?**	
**What are your treatment recommendations at the present time and without further diagnostics (multiple choice possible)?**	☐ Resection (=OP). If yes, please specify (multiple selection):<br>☐ preferred laparoscopic<br>☐ preferred Laparotomy<br>☐ lymphnode dissection<br>☐ Biopsy<br>☐ preferred sonographic<br>☐ preferred Laparoscopic<br>☐ Watch and wait<br>☐ Others<br>Comments: ______________________
	☐ Chemotherapy (=CTX)<br>• Active substance:<br>• Dose:<br>• Number of cycles:<br>• Duration per cycles:
	☐ Radiotherapy (=RT)<br>• Type of radiation (photons, protons, etc.):<br>• Dose per Fraction:<br>• Fraction per day:<br>• Total dose:<br>• Technic:
	☐ Mitotane<br>• Target serum level:<br>• Starting Dose:<br>• Duration of treatment:
	☐ Targeted therapy (=TT)<br>• Active substance:<br>• Dose:<br>• Number of doses:
	☐ Best supportive care
	☐ Inclusion in the following study/Registry:

<table>
<tr><td></td><td colspan="10">☐ Other suggestions:</td></tr>
<tr><td>Multimodal therapy:

If you have recommended a multimodal therapy, please indicate the planned sequence</td><td colspan="10">- - -</td></tr>
<tr><td>What is your rationale for the procedure (free text) ?</td><td colspan="10"></td></tr>
<tr><td>What was the consensus of all parties involved in this decision? (from 1(low)-10 (high))</td><td>1
☐</td><td>2
☐</td><td>3
☐</td><td>4
☐</td><td>5
☐</td><td>6
☐</td><td>7
☐</td><td>8
☐</td><td>9
☐</td><td>10
☐</td></tr>
<tr><td>Best alternative procedure (free text) ?</td><td colspan="10"></td></tr>
</table>

**Specialities present at the tumour conference meeting (multiple choice possible)**	☐ General and visceral surgery	☐ Pediatric surgery
	☐ Radiotherapy	☐ ped. Oncology
	☐ Pathology	☐ adult Oncology
	☐ Radiology (incl. Neurorad.)	☐ Nuclearmedicine
	☐ ped. Endocrinology	☐ adult Endocrinology
	☐	☐
	☐	☐
	☐ Urology	☐
	☐ Psychooncology	☐ social workers

Case 5	
**Patient characteristics**	• Age 5 5/12<br>• Sex: female<br>• Family history: without events
**Diagnosis**	• Adrenal gland Tumor left
**Clinical Course**<br><br><br><br><br><br><br><br>**Questiom**	• Adrenal carcinoma left, stage IV<br>Infiltrating vena cava, thrombus right atrium<br>16x11x16mm initial tumor mass<br>Androgen production with clinical signs of precocious puberty<br>Pulmonary filiae<br><br>o Therapy 8 blocks of chemotherapy according to GPOH-MET registry (Cisplatin, Eto, Doxo) plus Mitotane<br><br>**Staging at the end of intensive chemotherapy- further treatment? Surgery? Immune therapy? Mitotane only?**
**Pathology**	Not done until now
**Radiology**	MRI: pictures are uploaded<br>CT Thorax: are uploaded
**Other diagnosis**	• Li-Fraumeni-Syndrome<br>• Art. Hypertonia<br>• AV-Block I°, QTc elongation mild
**Please enter your tumour conference recommendation as free text**	
**Further diagnostics necessary?**	☐ No<br><br>**☐ Yes (multiple choice is possible):**<br><br>☐ Repeat follow-up imaging ☐ PET-CT<br>☐ Biopsy ☐ others<br>☐ Genetics<br>☐ recommended<br>☐ obligatory<br>☐ not necessary<br><br>Comments (free text):
**Recommended endocrine workup (free text)**	
**What are your treatment recommendations at the present**	

<table>
<tr><td>time and without further diagnostics (free text)?</td><td></td></tr>
<tr><td rowspan="8">What are your treatment recommendations at the present time and without further diagnostics (multiple choice possible)?</td><td>☐ Resection (=OP). If yes, please specify (multiple selection):
☐ preferred laparoscopic
☐ preferred Laparotomy
☐ lymphnode dissection
☐ Biopsy
☐ preferred sonographic
☐ preferred Laparoscopic
☐ Watch and wait
☐ Others
Comments: ____________________</td></tr>
<tr><td>☐ Chemotherapy (=CTX)
• Active substance:
• Dose:
• Number of cycles:
• Duration per cycles:</td></tr>
<tr><td>☐ Radiotherapy (=RT)
• Type of radiation (photons, protons, etc.):
• Dose per Fraction:
• Fraction per day:
• Total dose:
• Technic:</td></tr>
<tr><td>☐ Mitotane
• Target serum level:
• Starting Dose:
• Duration of treatment:</td></tr>
<tr><td>☐ Targeted therapy (=TT)
• Active substance:
• Dose:
• Number of doses:</td></tr>
<tr><td>☐ Best supportive care</td></tr>
<tr><td>☐ Inclusion in the following study/Registry:</td></tr>
<tr><td>☐ Other suggestions:</td></tr>
<tr><td>Multimodal therapy:

If you have recommended a multimodal therapy, please</td><td>- - -</td></tr>
</table>

<table>
<tr><td>indicate the planned sequence</td><td colspan="2"></td></tr>
<tr><td>What is your rationale for the procedure (free text) ?</td><td colspan="2"></td></tr>
<tr><td>What was the consensus of all parties involved in this decision? (from 1(low)-10 (high))</td><td colspan="2">1 ☐ 2 ☐ 3 ☐ 4 ☐ 5 ☐ 6 ☐ 7 ☐ 8 ☐ 9 ☐ 10 ☐</td></tr>
<tr><td>Best alternative procedure (free text) ?</td><td colspan="2"></td></tr>
<tr><td rowspan="9">Specialities present at the tumour conference meeting (multiple choice possible)</td><td>☐ General and visceral surgery</td><td>☐ Pediatric surgery</td></tr>
<tr><td>☐ Radiotherapy</td><td>☐ ped. Oncology</td></tr>
<tr><td>☐ Pathology</td><td>☐ adult Oncology</td></tr>
<tr><td>☐ Radiology (incl. Neurorad.)</td><td>☐ Nuclearmedicine</td></tr>
<tr><td>☐ ped. Endocrinology</td><td>☐ adult Endocrinology</td></tr>
<tr><td>☐</td><td>☐</td></tr>
<tr><td>☐</td><td>☐</td></tr>
<tr><td>☐ Urology</td><td>☐</td></tr>
<tr><td>☐ Psychooncology</td><td>☐ social workers</td></tr>
</table>

Thank you!!!
